# EDTA/gelatin zymography method to identify C1s versus activated MMP‐9 in plasma and immune complexes of patients with systemic lupus erythematosus

**DOI:** 10.1111/jcmm.13962

**Published:** 2018-10-24

**Authors:** Estefania Ugarte‐Berzal, Erik Martens, Lise Boon, Jennifer Vandooren, Daniel Blockmans, Paul Proost, Ghislain Opdenakker

**Affiliations:** ^1^ Laboratory of Immunobiology Department of Microbiology and Immunology Rega Institute for Medical Research University of Leuven KU Leuven Leuven Belgium; ^2^ Department of General Internal Medicine University Hospital Gasthuisberg KU Leuven Leuven Belgium; ^3^ Laboratory of Molecular Immunology Department of Microbiology and Immunology Rega Institute for Medical Research KU Leuven Leuven Belgium

**Keywords:** complement system subunit C1s, EDTA/gelatin zymography, SLE marker, systemic lupus erythematosus

## Abstract

Gelatin zymography analysis is a sensitive method and commonly used to characterize and quantify the presence of the gelatinases (MMP‐2 and MMP‐9) in biological samples. In human plasma samples from healthy controls and systemic lupus erythematosus (SLE) patients, we observed a gelatinolytic molecule at 80 kDa, suggestive for activated human MMP‐9. However, by developing and using the EDTA/gelatin zymography method and after purification of the 80 kDa entity, we proved that this molecule was the C1s subunit of the complement system. The zymolytic capacity of C1s was validated and found to be enhanced, in the absence of calcium and in the presence of EDTA. Our findings indicate that for correct identification of gelatinolytic proteins in complex biological samples the use of EDTA/gelatin zymography for enzyme development is advised. In addition, by quantification of EDTA/gelatin zymography analysis and ELISA, we observed that the levels of C1s were higher in plasma and immune complexes of SLE patients than of healthy individuals. Therefore, our data imply that C1s may become a marker for the diagnosis of SLE.

## 1 INTRODUCTION

Systemic lupus erythematosus (SLE) is a complex and heterogeneous autoimmune disease that affects many organs, including skin, joints, kidneys, and central nervous system. SLE is characterized by high levels of autoantibodies and circulating immune complexes (IC), which become deposited in several organs, thereby causing tissue damage.^1^ Most of the autoantibodies present in SLE are against nuclear proteins, as well as against DNA and RNA. Presently, more than 180 different types of autoantibodies have been already described in the plasma of SLE patients.^2^

The development of SLE is complex and requires the dysregulation of several processes. Defects of B and T lymphocytes are reported. Specifically, the negative selection of autoreactive B and T cells is disrupted in SLE patients. Moreover, SLE patients present high apoptotic and necrotic cell rates, in comparison with healthy controls. In addition, inefficient clearances of these dead cells by the complement system and by macrophages take place. This pathogenic context generates increased autoantibody levels against ubiquitous molecules and consequently IC formation.^1^, ^3^ Furthermore, several other molecules play a role in the progression of SLE diseases and these include many inflammatory mediators such as cytokines, chemokines and matrix metalloproteinases (MMPs).^4^, ^5^, ^6^, ^7^

The complement system consists of more than 30 proteins that are soluble or covalently attached to target (cell) surfaces.^8^ It regulates many biological functions such as activation of B lymphocytes, degranulation of mast cells and granulocytes, solubilization and clearance of IC and membrane attack‐complex‐associated cell lysis.^8^ Although the activation of the complement system is important to clear infections, it also contributes to the inflammatory response triggered by IC deposition in tissues in autoimmune diseases, thereby playing an important role in SLE.^9^, ^10^, ^11^

The classical complement activation pathway forms a bridge between adaptive and innate immunity. The first component C1q binds to antibodies within IC. C1q binds with two zymogens of C1r and two of C1s. Once the C1q is bound to IC or pathogens, the complex undergoes a conformational change, which leads to C1r activation, that immediately activates C1s, generating an active serine protease.^12^ Thereafter, C1s activates the next two components of the classical pathway C4 and C2, which at the same time will cleave C3 into C3a and C3b. Most of these complement components are dysregulated in SLE.^9^ Genetic deficiencies of C1q, C1r, C1s, C2, C4, and C3 are associated with SLE development. In these cases, the disease usually starts early in life, has an equal incidence among males and females and is characterized by multi‐organ involvement with glomerulonephritis in approximately 30% of the cases.^9^ The levels of complement molecules and their functional activities in serum are used as markers of disease activity in SLE. During periods of active disease, serum complement activity is usually reduced.

Autoantibodies against complement system components are detected in SLE patients.^13^, ^14^ Interestingly, autoantibodies against C1s are present in SLE patients and enhance the proteolysis/enzymatic capacity of C1s, thereby inducing C4 activation and the complement system.^14^

Matrix metallopeptidase 9 (MMP‐9), also called gelatinase B, mediates vital physiological processes. The dysregulation of this molecule is associated with several pathological conditions, including SLE,^15^ although its role in SLE is still unclear. The data in the literature are not consistent, because researchers utilized different sources and techniques to study MMP‐9 in relation with SLE. Some groups measured MMP‐9 levels in plasma or serum samples by ELISA,^16^, ^17^ whereas others studied the levels of MMP‐9 secreted by peripheral blood mononuclear cells.^18^ In addition, others measured MMP‐9 levels by gelatin zymography.^19^, ^20^

Because of discrepancies about the pathogenic role of MMP‐9 in SLE and the finding of opposing results, we hypothesized that some results of the used analytical methods might have been misinterpreted. Gelatin zymography is the most sensitive method to detect gelatinases. However, other proteases, including the serine protease trypsin, cleave denatured collagen or gelatin.^21^ In zymography analysis, information about molecular weights is used to identify MMP‐9 forms, including multimeric MMP‐9, NGAL‐MMP‐9 complexes, proMMP‐9, activated MMP‐9 (actMMP‐9) and differences in glycosylation. Other proteases, co‐migrating with such MMP‐9 forms, maybe misinterpreted as MMP‐9 forms. We analyzed the gelatinolytic banding patterns of plasma samples from healthy controls and patients suffering from SLE. Surprisingly, by introduction of EDTA/gelatin zymography, we discovered the presence of complement C1s zymolysis at around 80 kDa, which might be misinterpreted as actMMP‐9. We also provided the methodology to detect C1s and to discriminate it from actMMP‐9. We observed that C1s levels were significantly higher in plasma and IC from SLE patients than in control samples and this finding might be relevant for SLE diagnosis and staging.

## 2 MATERIALS AND METHODS

### 2.1 Reagents

Antibodies against complement components C1s (Sheep Anti‐human C1s, AF2060, and mouse anti‐human C1s MAB2060) and C1r antibodies (AF1807‐SP) and antibodies recognizing human MMP‐9 (AF911) and human MMP‐2 (AF902) were purchased from R&D Systems (Minneapolis, MN, USA). HRP‐conjugated antibodies were purchased from Jackson Human Research (Cambridgeshire, UK). Recombinant C1s (H00000716‐P01) was from Abnova (Taipei City, Taiwan). Gelatin from bovine skin, type B (G9391) for zymography analysis was from Sigma (Sigma‐Aldrich, St Louis, MO, USA). EDTA, protein G‐Sepharose 4 Fast Flow (71‐7083‐00) and gelatin‐Sepharose 4B (17‐0956‐01) were purchased from GE Healthcare (Chicago, IL, USA), Coomassie brilliant blue (B0149) was from Sigma. EDTA (VWR‐ 20302.260) was from BDH‐chemicals (Brooklyn, NY, USA).

### 2.2 Plasma samples

Blood samples from healthy controls and SLE patients were centrifuged at RT and 1500 *g* for 5 minutes. The resulting supernatants were used as plasma samples. These were collected and stored at −80°C in several small aliquots for optimal conservation. All donors gave written consent and all procedures were according to the terms of the declaration of Helsinki and following Belgian and European legislation. In Table S1 information about the SLE patients is provided. All patients suffered from clinical SLE symptoms and were under various anti‐inflammatory treatments at sampling. The control samples came from healthy donors (3 males, 17 females).

### 2.3 Gelatin zymography

The proteins were separated in 7.5% polyacrylamide gels containing sodium dodecyl sulfate (SDS) and 1 mg/ml gelatin.^21^ After electrophoresis, the gels were washed with 2.5% Triton X100 to remove the SDS and incubated overnight in 50 m mol L^−1^ Tris‐HCl pH 7.5, 10 m mol L^−1^ CaCl_2_ at 37°C for the development of zymolytic bands. To inhibit the metalloproteases activity during enzyme development, the gels were incubated with 10 m mol L^−1^ EDTA. Protease bands were detected by absence of Coomassie Brilliant Blue staining of digested gelatin. Recombinant proMMP‐9 standard mixture (including the delection mutant MMP‐9ΔOGHem, which lacks the O‐glycosylated and hemopexin domains) was used in each gel as a control. The different protease bands were qualitatively and quantitatively analyzed with ImageJ TL software.^21^

### 2.4 Anion exchange chromatography

For the purification of the 80 kDa zymolytic proteins a two‐step purification strategy was used, followed by gelatin‐zymography and Maldi/TOF/MS analysis after tryptic in‐gel digest of the target proteins.

A first step anion exchange chromatography was performed with 20 ml human plasma from healthy donors. A 300 ml Q‐Sepharose fast flow column (GE Healthcare) was equilibrated with 30 m mol L^−1^ Tris/HCl pH 8.9 and, after loading with the sample (1/5 diluted in equilibration buffer), the column was eluted with a linear gradient from 0 mol L^−1^ NaCl to 0.5 mol L^−1^ NaCl over 5 column volumes. The fractions were analyzed by zymography in the presence and absence of 10 m mol L^−1^ EDTA. The fractions (numbers 59‐72), containing the EDTA‐resistant zymolytic protein(s), were concentrated and dialyzed against 20 m mol L^−1^ piperazine pH 6.3 with 6 mol L^−1^ urea. The urea was added to prevent non‐covalent protein interactions.

The second purification step was an anion exchange a low pH under denaturing conditions on a 20 ml HiLoad Q Sepharose FF column (GE Healthcare). The concentrated and dialyzed fractions of the first purification step were loaded on this column. The column was then eluted with a two‐step gradient. The first step was a linear gradient from 0 mol L^−1^ NaCl to 0.5 mol L^−1^ NaCl in 20 m mol L^−1^ piperazine pH 6.3 containing 6 mol L^−1^ urea over 15 column volumes and the second elution was with a linear gradient from 0.5 mol L^−1^ NaCl to 1 mol L^−1^ NaCl in the same buffer over 3 column volumes.

In the EDTA/gelatin zymography analysis, the two visualized bands contained the target protein(s). In the center of the outlined zone, faintly stained bands were seen. The bands were carefully sliced out and analyzed by nanoLC/TOF/MS analysis after in‐gel trypsin digests.

### 2.5 nanoLC/TOF/MS and protein identification

The nanoLC/TOF/MS and protein identification were done by Alphalyse (Alphalyse A/S, 5220 Odense, Denmark). Briefly, the protein samples were reduced and alkylated with iodoacetamide, ie, carbamidomethylated, and subsequently digested with trypsin. The resulting peptides were concentrated by Speed Vac lyophilization and redissolved for injection on a Dionex nano‐LC system and MS/MS analysis on a Bruker Maxis Impact QTOF instrument. The MS/MS spectra were used for Mascot database searching. The data were searched against in‐house‐Alphalyse protein databases downloaded from UniProt and NCBI containing more than 80 million known non‐redundant protein sequences. The Mascot software found all the matching proteins in the database by their peptide masses and peptide fragment masses. The protein identification was based on a probability‐scoring algorithm (www.matrixscience.com) and the best matching protein was C1s. A positive protein identification was considered when at least two peptides had an MS‐ions score above 35 or if a protein under 20 kDa had one peptide with an MS‐ions score above 50.

### 2.6 Immunoprecipitation and Western blot analysis

For precipitation of IC, 50 μl of plasma sample was incubated with protein G‐Sepharose for 1 hour at RT. The pellets containing the IC and free antibodies were washed with PBS buffer and proteins extracted by addition of Laemmli buffer.

For Western blot analysis, the samples were resolved by and transferred to PVDF membranes (Bio‐Rad Laboratories, Hercules, CA). After protein transfer the membranes were blocked with 5% BSA for 1 hour and incubated (4°C, 16 hours) with primary antibodies, followed by incubation for 1 hour at RT with HRP‐labelled secondary antibodies. Protein bands were developed using the enhanced chemiluminiscence detection method (Super Signal West Pico Plus 34557, Thermo Scientific, MA, USA).

### 2.7 Enzyme‐linked immunosorbent assays for C1s

For ELISAs, 96 well plates were coated with 2 μg of sheep anti‐human C1s antibody. After washing and blocking the plates, the studied samples were added and incubated overnight at 4°C. The plates were then washed and the samples were incubated with the C1s detection antibody (of mouse origin). After washing, a specific secondary anti‐mouse IgG‐HRP‐conjugated antibody was added and incubated in the plate for 1 hour at RT. Finally, the plate was washed and developed.

### 2.8 Statistical analyses

Statistical analysis were performed using GraphPad Prism 6 software. Significant differences between experiments were evaluated using a non‐parametric ANOVA Kruskal‐Wallis test. All *P* values of 0.05 or less were considered significant.

## 3 RESULTS

### 3.1 Identification of a gelatinolytic 80 kDa molecule in human plasma samples, which is not MMP‐9

**Figure 1:**
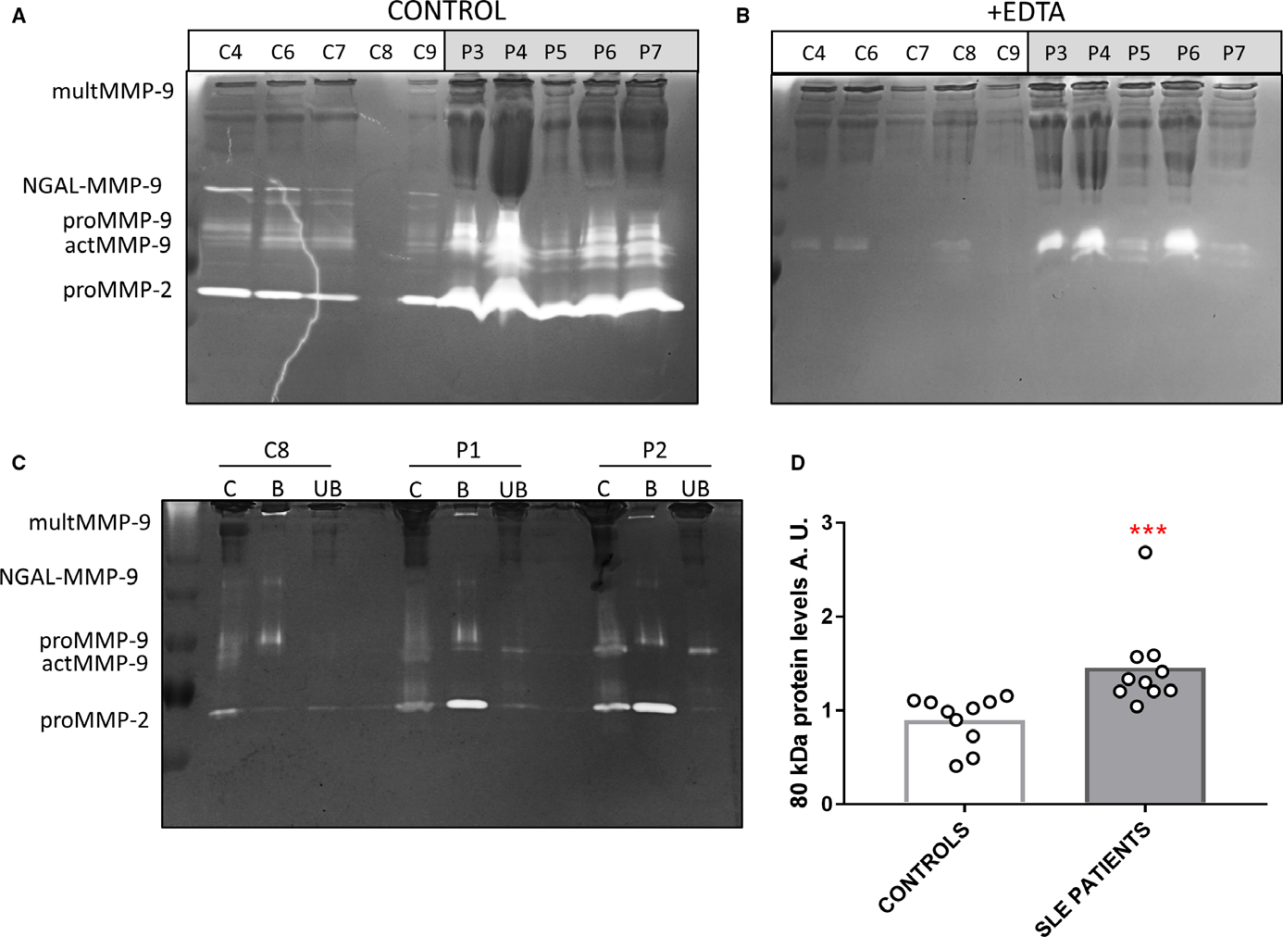
Identification of an unknown protease with higher levels in SLE versus control plasma: A, 2 μl of plasma sample from healthy controls (C4‐C9) and SLE patients (P3‐P7) was analyzed by gelatin zymography. B, Analysis of similar aliquots as in panel A in the presence of 10 m mol L^−1^ EDTA. C, 50 μl of plasma samples from SLE patients and healthy controls were incubated with gelatin‐Sepharose. The unbound (UB) and bound (B) fractions were analyzed by gelatin zymography. D, Quantification of the unknown‐protein bands by gelatin zymography analysis in the presence of 10 m mol L^−1^ EDTA (panel B). *P* values were determined by ANOVA Kruskal‐Wallis test, ***P < 0.001. A.U.: Arbitrary units. Dots indicate samples from individual subjects

With the intention to study the levels of MMP‐9 in plasma samples from SLE patients, we performed gelatin zymography analysis on 2 μl of plasma samples. Gelatin zymography revealed several gelatinolytic proteins of different molecular weights (Figure 1A). High molecular weights bands (>250 kDa) represented MMP‐9 trimers and complexes of MMP‐9/‐2 with α2‐macroglobulin. The NGAL‐MMP‐9 complex was detected at 150 kDa and proMMP‐2 at 72 kDa. In the electrophoretic mobility range of proMMP‐9 and its activated (actMMP‐9) forms, approximately between 95 and 80 kDa, respectively, a collection of zymolytic bands was visible. To distinguish the presence of MMP‐2 or MMP‐9 from non‐metalloproteases, we incubated gelatin zymography gels in the presence of EDTA to inhibit metalloproteinase activity (Figure 1B). A major 80 kDa band was not inactivated by EDTA. This molecule was barely visible in normal plasma, but was abundant in SLE patient plasma samples (Figure 1B). The dark protein bands observed in the gels mainly corresponded to immunoglobulins and IC staining with Coomassie blue. To confirm the different nature of this EDTA‐resistant enzyme, we examined its gelatin binding property to gelatin‐Sepharose (Figure 1C). As expected, proMMP‐9 (92 kDa), actMMP‐9 (80 kDa) and MMP‐2 (72 kDa) were detected in the bound fractions. However, in the unbound fractions, a protease with a molecular weight, which might be misinterpreted as the activated form of MMP‐9 (80 kDa), was detected (Figure 1C) in SLE patients P1 and P2. Quantification of this specific zymolysis, in the presence of EDTA, showed significantly increased levels of this protease in plasma samples of the SLE patients analyzed compared with the healthy controls (Figure 1D).

### 3.2 Purification and identification of the 80 kDa protease

Since the levels of the unknown 80 kDa protease detected upon zymography were significantly higher in plasma from SLE patients than in healthy control samples, we hypothesized that this molecule might play a role in SLE. In an attempt to purify and identify the unknown protease, we performed anion exchange chromatography (Material and Methods). All the obtained fractions were analyzed by gelatin zymography (Figure S1A). However, the gelatinolytic bands did not disclose any information whether the 80 kDa protease was activated MMP‐9. Hence, selected fractions were additionally analyzed in the presence of EDTA to distinguish the fractions containing the unknown protease from those with MMP‐9. In Figure S1B, we showed that the unknown protease(s) were detected, in the presence of EDTA, in fractions 60‐70. These fractions were pooled and subjected to a second anion exchange chromatography (see Materials and Methods) (Figure S1C). By zymography analysis, we showed considerable levels of the unknown protease(s) in the fractions 39, 40 and 41. The goal of performing several anion exchange chromatography steps was to purify the unknown protein to be able to identify it by trypsin digestion followed by nanoLC‐MS/MS peptide sequencing and database search (Alphalyse). The purity of the fractions 39, 40, and 41 was analyzed by Coomassie blue staining of proteins (Figure S2). The bulk of proteins between 70 and 100 kDa (orange square), where the unknown‐protein was located, were mostly cleared in this molecular weight range. Consequently, we proceeded to do a zymography in the presence of EDTA. At the center of the indicated zone, the protein(s), responsible for this gelatinolytic activity, were present and these were not accompanied by many other abundant proteins. This center was carefully sliced out of the gel for tryptic digestion followed by nanoLC‐MS/MS peptide sequencing and database search (Alphalyse).

Several peptides from different proteins were detected by the database search (Figure S2), and on the bases of the molecular sizes, amounts of detected peptides and characteristics of the proteins, we suggested that C1s was the unknown zymolytic protease.

### 3.3 Validation of C1s as identified gelatinolytic protein

**Figure 2:**
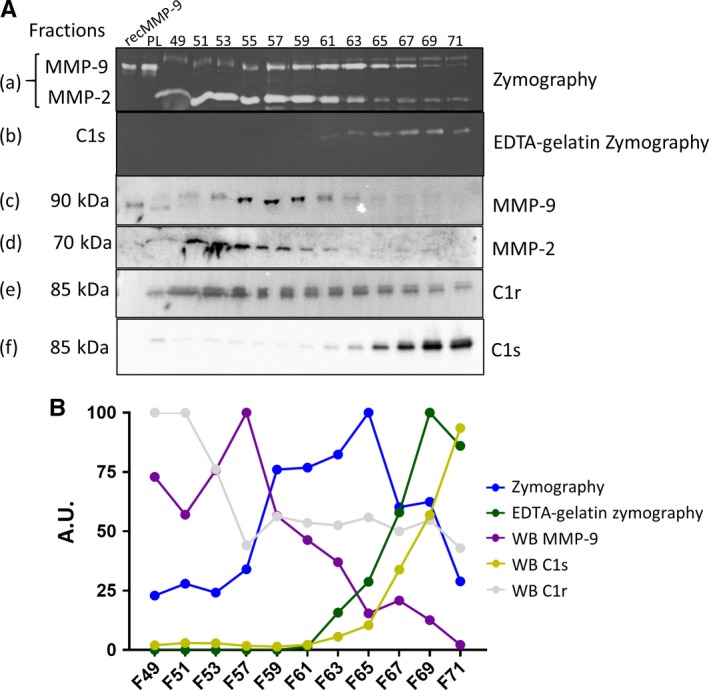
Corroboration of C1s as the unknown protease: A, similar volumes of the selected fractions from the first anion exchange chromatography analyses were tested by gelatin zymography in the absence (a) or presence (b) of 10 m mol L^−1^ EDTA, or by Western blot analysis, to identify MMP‐9 (c), MMP‐2 (d), C1r (e), and C1s (f). B, Quantification of the bands from panel A. PL: plasma, WB: Western blot, F: fraction, A.U.: arbitrary units

After proteomic analysis of C1s as the putative protease, we proceeded to corroborate this finding. Similar volumes of the fractions from the first anion exchange chromatography elution (30 μl) were analyzed by gelatinzymography in the presence or absence of EDTA. In parallel, the presence of MMP‐9, MMP‐2, C1s, and C1r were analyzed by Western blot in the same fractions (Figure 2A) (equal volumes of 50 μl for all the fractions). All the bands (except MMP‐2) were quantified as is shown in Figure 2B. The results from the Western blot analysis of C1s showed that this protease was present in the same fractions (mainly fractions 63‐71) in which we observed gelatinolytic activity in the presence of EDTA. In contrast, the MMP‐9 signal in the Western blot (mainly fractions 53‐71) did not perfectly correlate with the zymolytic activity detected in the standard gelatin zymography. In the case of MMP‐2, Western blot immunoreactivity and gelatinolytic bands detected at 70 kDa were present in similar fractions. C1r levels were also studied, for the reason that it is another protease that forms a complex with C1q and C1s and because C1r has considerable homology with C1s. However, C1r was distributed differently than the EDTA‐resistant protease in the Western blot analysis (Figure 2B). We herewith provided a quality control of the used antibodies and complementary information excluding C1r and reinforcing C1s as the unknown EDTA‐resistant protease. In conclusion, these analyses validated that C1s was the searched molecule and that it possesses newly discovered gelatinolytic activity. The co‐migration of human C1s with human MMP‐9 upon classical gelatin zymography stimulated a critical re‐appraisal of previously published data.

### 3.4 Characterization and optimization of the detection of C1s in zymography analysis

**Figure 3:**
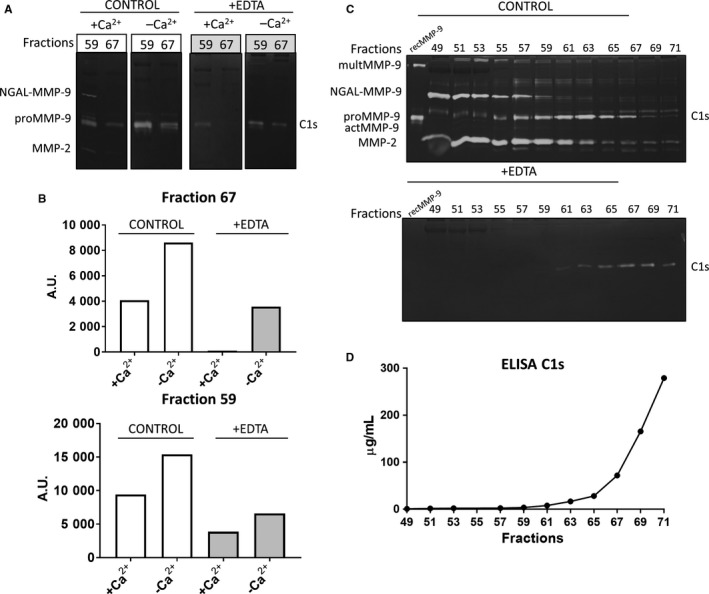
Characterization and optimization of C1s detection by zymography: A, Fractions 59 and 67 (30 μl) from the first anion exchange were analyzed by gelatin zymography in the presence or absence of 10 m mol L^−1^ of EDTA and/or Ca^2+^. B, Example of quantification of the gelatinolytic bands from panel A. C, Zymography analysis of selected fractions from the anion exchange in the absence (CONTROL) or presence of 10 m mol L^−1^ EDTA (+EDTA). D, The levels of C1s were measured by ELISA in the same fractions, as analyzed in panel C. A.U.: arbitrary units

After corroboration that C1s was the EDTA‐resistant protease detected at the molecular weight of 80 kDa in gelatin zymography, besides actMMP‐9, we optimized the detection of C1s molecule with the EDTA/gelatin zymography technique. We studied the gelatinolytic capacity of C1s in the presence or absence of EDTA and/or Ca^2+^. As expected, in the presence of EDTA all the signal from metalloproteases was inhibited and solely the proteolytic C1s band remained visible. Interestingly, the signal from C1s in gelatin zymography was also enhanced in the absence of Ca^2+^ (Figure 3A,B).

To define the minimal detectable amount of C1s by EDTA‐gelatin zymography we quantified the C1s concentration in the fractions of the first anion exchange chromatography by a sandwich ELISA. According to the ELISA data, the fractions from 63 until 71 contained increasing amounts of C1s (Figure 3C,D). All these fractions showed the presence of the 80 kDa zymolytic band in EDTA‐gelatin zymography (Figure 3C). The C1s concentration of fraction 63 was 20 μg/ml, 30 μl of this fraction was charged in the zymography analysis. A fair conclusion thus was that samples containing more than 600 ng of C1s created a clearly detectable band in gelatin zymography analysis.

### 3.5 Detection of C1s in plasma samples and IC of SLE patients

**Figure 4:**
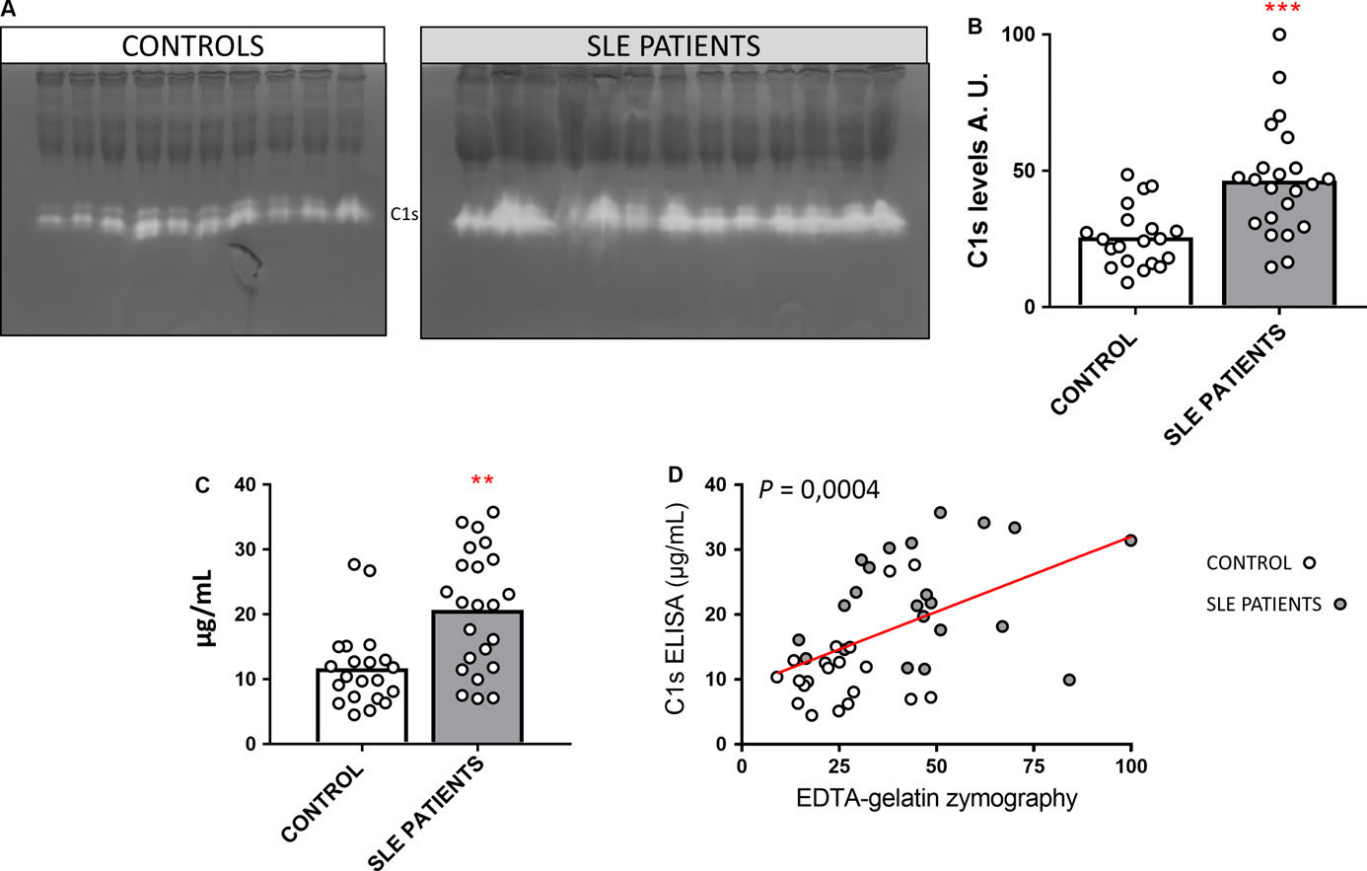
Analysis of C1s levels in plasma samples by EDTA‐gelatin zymography and ELISA: A, Representative pictures of two EDTA‐gelatin zymography gels from the analysis of plasma samples from healthy controls and SLE patients. B, Quantification of the gelatinolytic protein levels after EDTA‐gelatin zymography of 20 healthy control and 22 SLE patient plasma samples. *P* values were determined by ANOVA Kruskal‐Wallis test, ***P < 0.001. C, The histograms represent the mean concentrations of C1s from 22 plasma samples of SLE patients and 20 healthy individuals, as measured by ELISA. *P* values were determined by ANOVA Kruskal‐Wallis test, **P < 0.01. D, Correlation analysis of the data from the EDTA‐gelatin zymography in panel B and C1s ELISA from panel C

To verify the higher levels of C1s in plasma samples of SLE patients, we analyzed the levels of C1s in a cohort of plasma samples from healthy individuals and SLE patients by quantification of the bands obtained with EDTA‐gelatin zymography (Figure 4A,B). In parallel, we performed ELISA to detect C1s in the same plasma samples (Figure 4C). Both complementary analytical techniques gave similar results (*P* = 0.0004) (Figure 4D). Therefore, our results corroborated the finding (Figure 1) of significantly higher levels of C1s in plasma of SLE patients (n = 22) versus healthy controls (n = 20) (Figure 4).

**Figure 5:**
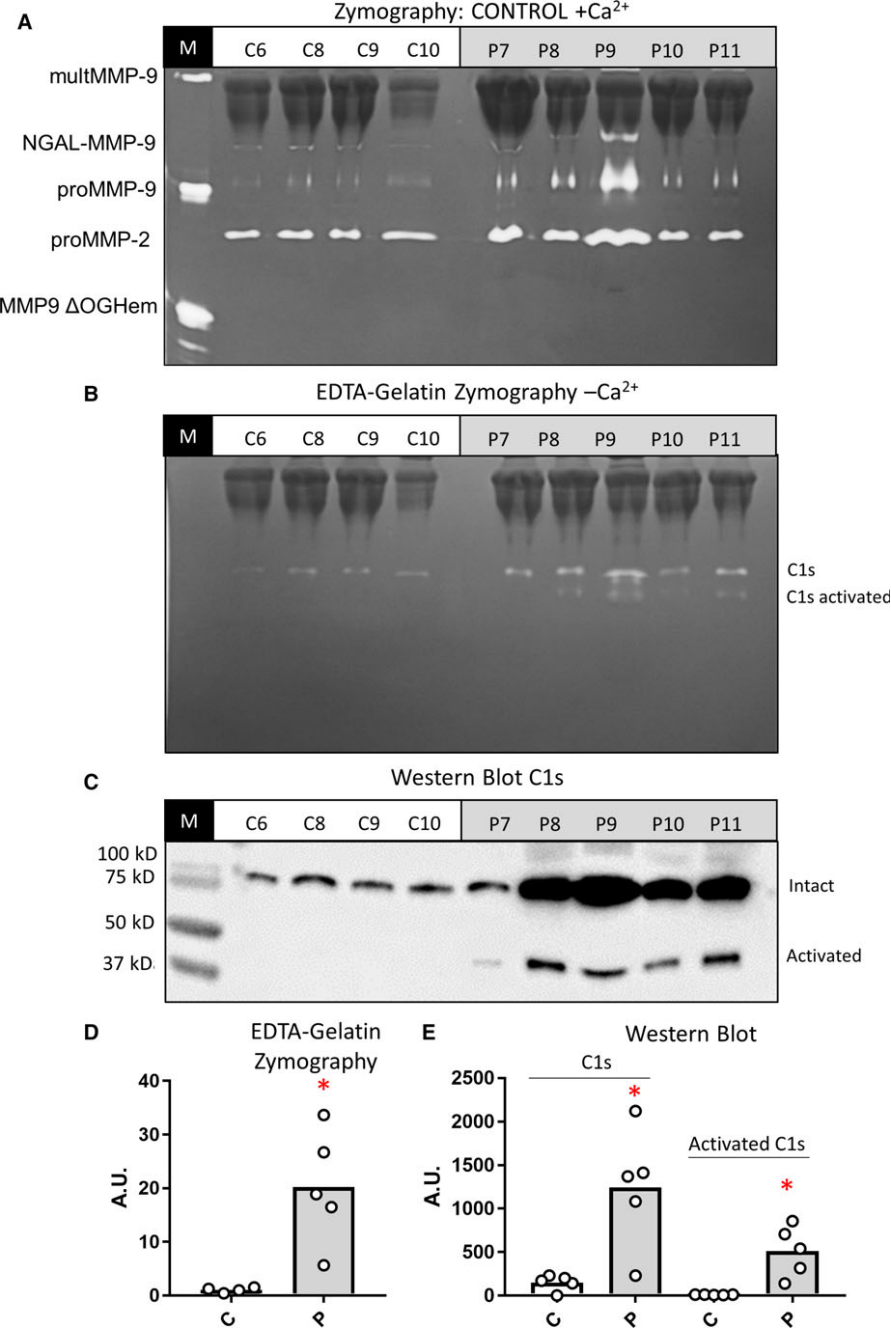
Study of the presence of C1s in IC: (A and B), 50 μl volumes of plasma samples from healthy controls and SLE patients were incubated with protein‐G‐Sepharose to precipitate the IgG and IgG‐IC. The bound fractions were analyzed by gelatin zymography in the absence (A) or presence (B) of 10 m mol L^−1^ EDTA. C, Western blot analysis of the same fractions shown in panels A and B for the detection of C1s. D, Quantification of the bands at 90‐80 kDa detected in the zymography from panel B. E, Quantification of the Western blot analysis. *P* values were determined by ANOVA Kruskal‐Wallis test, *P < 0.05

It is known that C1 binds to IC. Therefore, we studied if the presence of C1s in IC might be analyzed by gelatin zymography. Since the levels of IC in SLE plasma are high, we compared plasma samples of patients suffering from SLE and healthy individuals. First, we purified the IgG and IgG‐IC by protein G‐Sepharose precipitation. The results showed that plasma of the studied patients suffering from SLE had higher levels of C1s in the protein G‐bound fractions, thus in the IC (Figure 5A,B). Additionally, we showed that the levels of C1s had a trend towards higher levels in the unbound fractions, confirming that the levels C1s were higher in the plasma from the SLE patients analyzed compared with the healthy control samples (Figure S3).

To confirm the presence of C1s in IC, we analyzed the presence of C1s by Western blot (Figure 5C). The quantification of the EDTA‐zymography analysis (Figure 5D) and the Western blot for C1s (Figure 5E) corroborated the increased levels of C1s in IC of the SLE patients versus controls. In addition, in the IC from SLE patients the activated band of C1s was also detected, both upon EDTA‐gelatin zymography and Western blot analysis, in line with an activated state of the classical complement pathway.

## 4 DISCUSSION

In this manuscript, we revealed that (a) previously annotated human activated MMP‐9 in plasma or serum samples may be C1s; (b) with the use of EDTA/gelatin zymography these entities were identified and discriminated; (c) the EDTA/gelatin zymography technique was optimal in the absence of Ca^2+^ and (d) we discovered that the levels of C1s were higher in plasma and IC of the SLE patients analyzed versus control individuals.

Gelatin zymography assays are commonly used to study gelatinases.^21^ However, analysis and interpretation of gelatin zymography data from complex sources, like plasma or tissue extracts, may be a difficult subject, since not only MMP‐2 and MMP‐9 degrade gelatin, but also other proteases such as neutrophil elastase or trypsin. Consequently, a critical analysis and complementary assays need to be performed to ratify the data obtained from gelatin zymography. MMP‐9, as other MMPs, is secreted as a zymogen and has to be activated to execute catalytic activity. The cysteine switch from the propeptide interacts with the catalytic Zn^2+^ in the active site, thereby keeping the proenzyme inactive.^22^ Hence, proMMP‐9 is often activated by cleavage of the propeptide, which may be accomplished by proteolytic activators such as kalikrein, plasmin, neutrophil elastase or active MMP‐3. This proteolytic removal of the propeptide shifts the molecular weight of human MMP‐9 from about 90 to 80 kDa. Both entities, proMMP‐9 and actMMP‐9 are detectable in gelatin zymography.^21^

The gelatin zymography from plasma samples in Figure 1A showed several bands besides the expected NGAL‐MMP‐9 complex, proMMP‐9 and MMP‐2. Without further analysis of the zymography, in most clinical and basic research literature such bands have been interpreted as different posttranslational modification forms (eg, glycosylation) of MMP‐9 or actMMP‐9. However, in the presence of EDTA, the signals derived from metalloproteinases are eliminated. Here we discovered that a clear band around 80 kDa persisted upon zymography analysis and was significantly increased in the plasma samples from the SLE patients studied versus controls. With the use of gelatin‐Sepharose precipitation we corroborated the finding that C1s was not a classical gelatinase since only MMP‐9 and MMP‐2 bound to gelatin through their fibronectin domains. Therefore, we coin the complementary technique EDTA/gelatin zymography for the detection of C1s.With the intention to identify the unknown protease, subsequent anion exchange chromatography purification steps were used for obtaining a relatively pure protein preparation. Protein identification by nanoLC‐MS/MS peptide sequencing and database searches provided a total of 16 proteins. C1s was the most abundant protein with a sequence coverage of 45.5%. Furthermore, the molecular weight of intact C1s corresponded with the signal detected on gelatin zymography (80 kDa) and the isoelectric point of the protein (pI = 4.86) corresponded with the elution profile in the anion exchange. In addition, using specific antibodies for C1s we confirmed its identification by Western blot. Our findings point out that for the interpretation of the gelatin zymography of complex samples at least an EDTA/gelatin zymography control is recommended.

The effect of EDTA in blinding MMPs is obvious. The positive effect of Ca^2+^ removal may be explained by conformational changes in C1s. It is known that C1s‐C1s dimers present a more compact conformation in the presence of Ca^2+^.^23^ Therefore, the absence of Ca^2+^ may promote C1s to adopt an open structure with optimized gelatinolytic capacity.

By EDTA/gelatin zymography we showed an increase in the levels of C1s in SLE plasma samples and SLE IC from the present patient cohort (n = 22). This result was corroborated by a specific ELISA for C1s. Therefore, our data suggest that the levels of C1s are higher in the plasma and IC of SLE patients. After future validations with additional patient and control cohorts, the measurement of C1s in plasma and IC may become a marker to assist with the diagnosis of SLE.

Patients with deficiencies in C1 components usually develop SLE: specifically, 91% of individuals with C1q deficiency and 60%‐66% of individuals with C1r‐C1s deficiencies.^9^, ^11^ The levels of C1s in SLE patients have not been studied in detail. De Bracco et al measured the levels of C1q, C1r and C1s and they made correlations between these 3 components of C1. They concluded that in the majority of SLE cases C1q, C1r and C1s presented a good correlation but also low C1q levels coexisted with high levels of C1s and C1r.^24^ Furthermore, in patients with lymphopenic hypogammaglobulinaemia the levels of C1s were normal, whereas C1q levels remained unusually low.^25^ All these data suggest that C1q and C1s are differently regulated in (auto)immune pathologies. The basis for this might be differences in the cell sources for C1s and C1q. Whereas almost all serum complement system proteins, including C1s and C1r, are synthetized in the liver,^26^ C1q is mostly secreted by macrophages and immature dendritic cells.^27^, ^28^, ^29^

Here we showed that C1s levels are considerable in the IC of the SLE plasma samples studied. Since the presence of C1s autoantibodies has been demonstrated in SLE plasma samples,^14^ we cannot exclude that some of the autoantibodies detected in the IC might be against C1s. Therefore, in the IC, we may be detecting the C1s attached to autoantibodies against C1s but also to C1q in IC.

SLE diagnosis is very challenging because no generally accepted diagnostic criteria exist. Usually, the diagnosis is based on clinical manifestations and laboratory tests, including detection of autoantibodies, functional tests and imaging analysis. Sometimes the diagnosis is made after exclusion of alternative diseases.^30^ Therefore, the discovery of new markers for better diagnosis of this disease is important. In this manuscript, we provide preliminary evidence that the levels of C1s in plasma and IC of SLE patients are significantly higher than in control individuals (n = 20) suggesting that C1s might become useful for diagnosis.

We conclude that EDTA/gelatin zymography is a relevant new exploratory method for detection of C1s in SLE and probably also in other pathologies.

## CONFLICT OF INTEREST

The authors declare to have no conflict of interest.

## AUTHOR CONTRIBUTIONS

EUB designed the study. EUB and EM designed and executed most of the experiments, LB and JV executed specific experiments. EUB and GO wrote the manuscript with input from all authors.

## Supporting information

Figure S1

Figure S2

Figure S3

Table S1
