# Supplementary figures and images for: EDTA/gelatin zymography method to identify C1s versus activated MMP‐9 in plasma and immune complexes of patients with systemic lupus erythematosus
Source: J Cell Mol Med. 2018 Oct 24;23(1):576–85. doi: 10.1111/jcmm.13962 (PMC6307758; doi:10.1111/jcmm.13962)

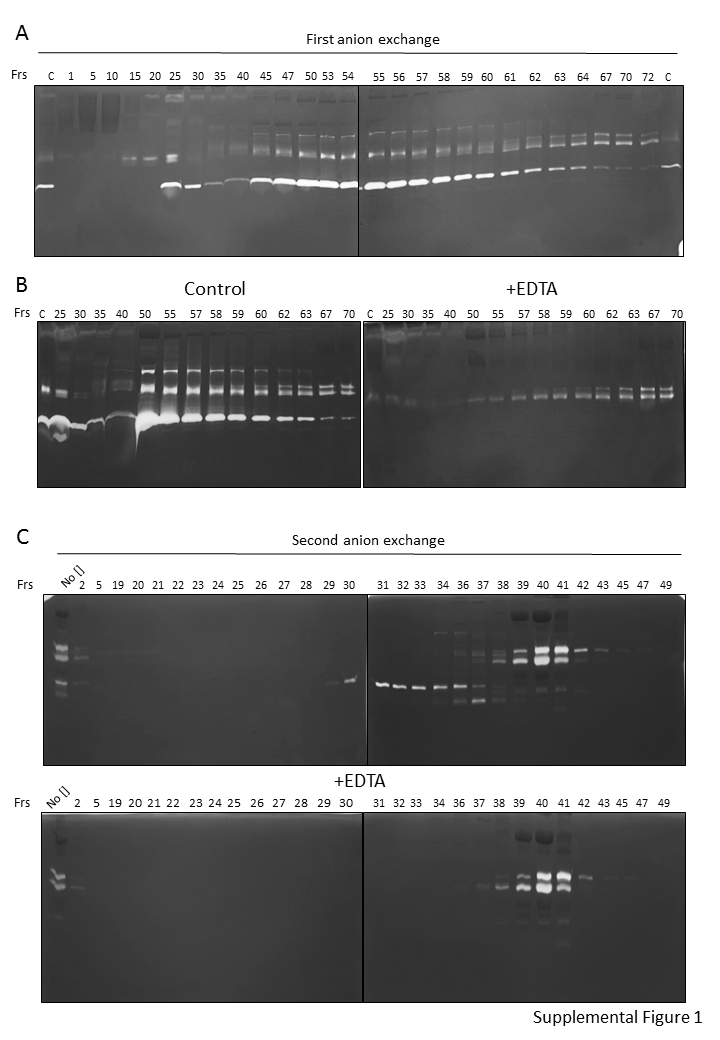

Supplement: Supplementary file 1 — Figure S1 [file JCMM-23-576-s001.tif]

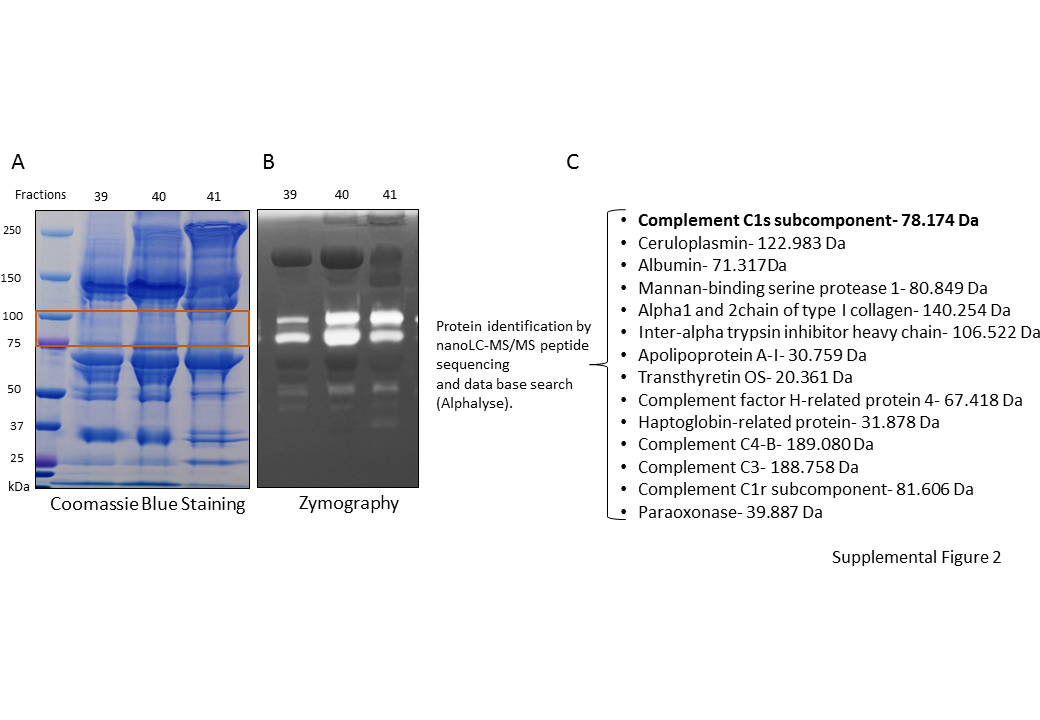

Supplement: Supplementary file 2 — Figure S2 [file JCMM-23-576-s002.tif]

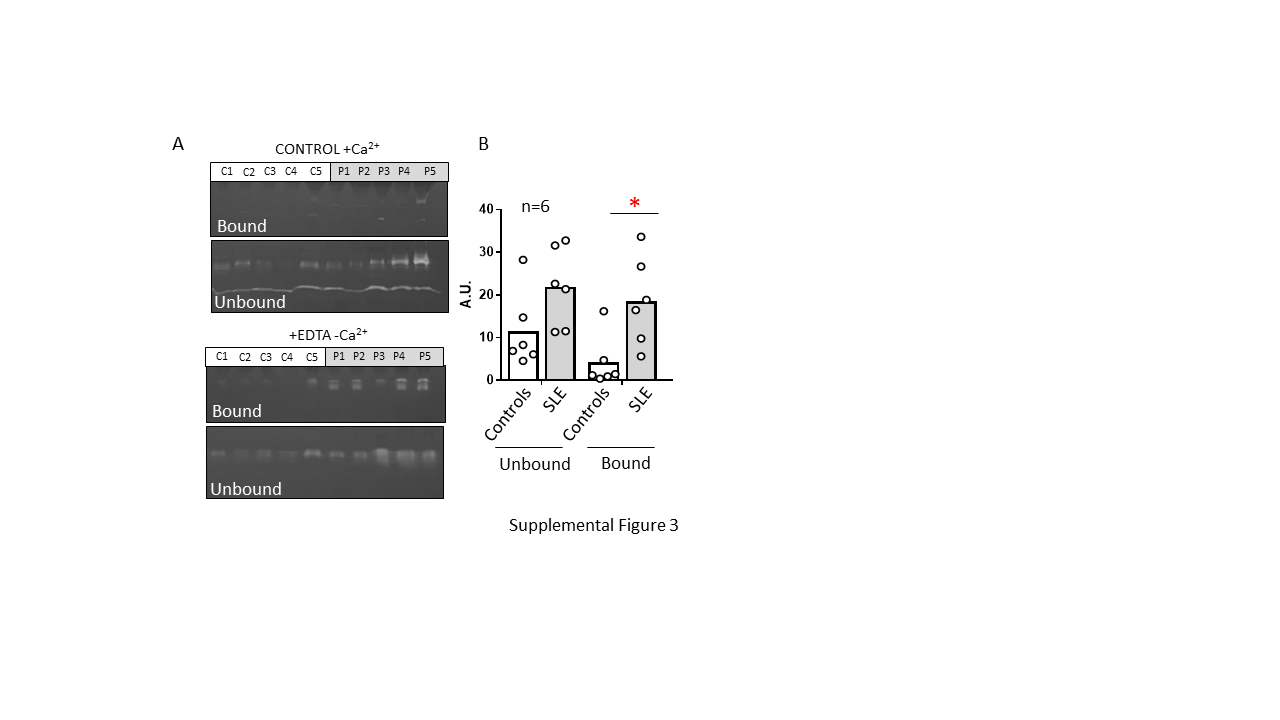

Supplement: Supplementary file 3 — Figure S3 [file JCMM-23-576-s003.tif]
