# Supplementary material for: EDTA/gelatin zymography method to identify C1s versus activated MMP‐9 in plasma and immune complexes of patients with systemic lupus erythematosus
Source: J Cell Mol Med. 2018 Oct 24;23(1):576–85. doi: 10.1111/jcmm.13962 (PMC6307758; doi:10.1111/jcmm.13962)
Supplement: Supplementary file 4 — Table S1 [file JCMM-23-576-s004.docx]

| **Patient** | **Gender** | **Pathology** | **Age**  **(years)** | **Treatment at sampling** | **Clinical disease activity** |
| --- | --- | --- | --- | --- | --- |
| **P1** | Female | SLE, Sjögren | 38 | Medrol, Plaquenil | non active |
| **P2** | Male | SLE, Sjögren | 43 | No documentation | No documentation |
| **P3** | Female | SLE | 37 | Plaquenil , Cellcept | active |
| **P4** | Female | SLE | 61 | Plaquenil, Aldactazine, Tildiem, Retard, Mobic | active |
| **P5** | Female | SLE, Sjögren | 50 | Plaquenil, Medrol, Sufrexal, Cacit D3, Pantazol | non active |
| **P6** | Female | SLE | 66 | Lasix , Aldactone , Zestril, Cardioaspirine, Nobiten, Staurodorm, Betaserc, Serenase, Dixarit | active |
| **P7** | Female | SLE | 73 | Lasix, Aldactone, Losec, Pravasine, Plaquenil, Asaflow, Emconcor, Burinex | non active |
| **P8** | Female | SLE | 46 | Pravasine | non active |
| **P9** | Female | SLE | 29 | Plaquenil | active |
| **P10** | Male | SLE | 25 | Medrol, Plaquenil, Diflucan | non active |
| **P11** | Male | SLE | 71 | No documentation | No documentation |
| **P12** | Female | SLE, Sjögren | 51 | Plavix, Emcoretic mitis, Lipitor | non active |
| **P13** | Male | SLE | 27 | Imuran, Natriumbicarbonaat, Calciumcarbonaat, Zestril, Zyloric, Aranesp, Selozok, Zantac, Foliumzuur | non active |
| **P14** | Female | SLE | 26 | Plaquenil | non active |
| **P15** | Female | SLE | 74 | Marcoumar, Steovit, Emconcor, Dafalgan, Promagnor, Actonel, Pantozol | non active |
| **P16** | Female | SLE | 76 | Medrol, Adalat oros, Plaquenil, Zolpidem | non active |
| **P17** | Female | SLE | 61 | Prednisone, Plaquenil a, Co-Lisinopril, Asaflow | non active |
| **P18** | Female | SLE | 52 | Medrol, Plaquenil, Ranitidine, Lorazepam, Pulmicort | active |
| **P19** | Female | SLE | 21 | Plaquenil, Triodene | non active |
| **P20** | Female | SLE | 37 | Medrol, Plaquenil, Asaflow | active |
| **P21** | Female | SLE | 15 | Zantac, Plaquenil, Medrol | non active |
| **P22** | Female | SLE | 24 | Cellcept, Lisinopril, Plaquenil | active |

**Supplemental Table 1:** Information of the SLE patient cohort of the study. The individual patient numbers (P1-P22) refer to the numbers used throughout the manuscript.
